# Dynamics of urinary and respiratory shedding of Severe acute respiratory syndrome virus 2 (SARS-CoV-2) RNA excludes urine as a relevant source of viral transmission

**DOI:** 10.1007/s15010-021-01724-4

**Published:** 2021-10-30

**Authors:** Jan-Niclas Mumm, Stephan Ledderose, Andreas Ostermann, Martina Rudelius, Johannes C. Hellmuth, Max Münchhoff, Dieter Munker, Clemens Scherer, Yannic Volz, Benedikt Ebner, Clemens Giessen-Jung, Christopher Lampert, Theresa Vilsmaier, Stephanie Schneider, Madeleine Gapp, Katrin Milger-Kneidinger, Jürgen Behr, Michael von Bergwelt-Baildon, Oliver T. Keppler, Christian Stief, Giuseppe Magistro, Michael Staehler, Severin Rodler

**Affiliations:** 1 Department of Urology, University Hospital, Ludwig Maximilian University of Munich (LMU), Marchioninistr. 15, 81377 Munich, Germany; 2 Institute of Pathology, University Hospital, Ludwig Maximilian University of Munich (LMU), Munich, Germany; 3 Max Von Pettenkofer Institute and Gene Center, Virology, National Reference Center for Retroviruses, Ludwig Maximilian University, Munich, Germany; 4 German Center for Infection Research, Partner Site Munich, Germany and Associated Partner Site Charité Berlin, Germany and Associated Partner Site Frankfurt, Munich, Germany; 5 Department of Medicine III and Comprehensive Cancer Center, University Hospital, Ludwig Maximilian University of Munich (LMU), Munich, Germany; 6 COVID-19 Registry of the LMU Munich (CORKUM), University Hospital, LMU Munich, Munich, Germany; 7 Department of Medicine V, Comprehensive Pneumology Center (CPC-M), Member of the German Center for Lung Research (DZL), University Hospital, Ludwig Maximilian University of Munich (LMU), Munich, Germany; 8 Department of Medicine I, University Hospital, LMU Munich, Munich, Germany; 9 Department of General, Visceral, and Transplant Surgery, Ludwig‐Maximilians‐University Munich, Munich, Germany; 10 Department of Gynecology, University Hospital, Ludwig Maximilian University of Munich (LMU), Munich, Germany

**Keywords:** Severe acute respiratory syndrome coronavirus 2, Urinary tract, COVID-19, Viral shedding

## Abstract

**Purpose:**

To investigate the expression of the receptor protein ACE-2 alongside the urinary tract, urinary shedding and urinary stability of SARS-CoV-2 RNA.

**Methods:**

Immunohistochemical staining was performed on tissue from urological surgery of 10 patients. Further, patients treated for coronavirus disease (COVID-19) at specialized care-units of a university hospital were assessed for detection of SARS-CoV-2 RNA in urinary samples via PCR, disease severity (WHO score), inflammatory response of patients. Finally, the stability of SARS-CoV-2 RNA in urine was analyzed.

**Results:**

High ACE-2 expression (3/3) was observed in the tubules of the kidney and prostate glands, moderate expression in urothelial cells of the bladder (0–2/3) and no expression in kidney glomeruli, muscularis of the bladder and stroma of the prostate (0/3). SARS-CoV-2 RNA was detected in 5/199 urine samples from 64 patients. Viral RNA was detected in the first urinary sample of sequential samples. Viral RNA load from other specimen as nasopharyngeal swabs (NPS) or endotracheal aspirates revealed higher levels than from urine. Detection of SARS-CoV-2 RNA in urine was not associated with impaired WHO score (median 5, range 3–8 vs median 4, range 1–8, p = 0.314), peak white blood cell count (median 24.1 × 1000/ml, range 5.19–48.1 versus median 11.9 × 1000/ml, range 2.9–60.3, *p* = 0.307), peak CRP (median 20.7 mg/dl, 4.2–40.2 versus median 11.9 mg/dl, range 0.1–51.9, *p* = 0.316) or peak IL-6 levels (median: 1442 ng/ml, range 26.7–3918 versus median 140 ng/ml, range 3.0–11,041, *p* = 0.099). SARS-CoV-2 RNA was stable under different storage conditions and after freeze–thaw cycles.

**Conclusions:**

SARS-CoV-2 RNA in the urine of COVID-19 patients occurs infrequently. The viral RNA load and dynamics of SARS-CoV-2 RNA shedding suggest no relevant route of transmission through the urinary tract.

**Supplementary Information:**

The online version contains supplementary material available at 10.1007/s15010-021-01724-4.

## Introduction

The *severe acute respiratory syndrome coronavirus 2* (SARS-CoV-2) impacts healthcare worldwide as the spread of the *coronavirus 2019 disease* (COVID-19) has been declared a pandemic by the WHO [1]. With the advance of pandemic, more is being understood about replication and transmission of this highly infectious virus [2]. ACE2 has been identified early as the main receptor of the spike protein of SARS-CoV-2 [3]. High expression levels are found in the alveolar epithelium of the lung explaining the affection of this tissue during infection [4]. Thereby, respiratory transmission appears to be the predominant way of transmission of SARS-CoV-2 [5]. However, as containment strategies seem to fail partially alternative ways of transmission have to be considered [6].

Urinary tract involvement in viral disease is mostly observed in immunocompromised patients. For example, BK polyomavirus and adenovirus are detected during hemorrhagic cystitis [7]. In recent viral epidemics, the transmission of viruses through genitourinary tissues has been detected. RNA of Zika virus for instance was detected in the urine of infected patients and infectious Zika virus was recovered from urine samples [8]. More dramatically, Ebola virus detected in the seminal fluid 531 days after onset of the disease caused a local outbreak in Guinea and Liberia [9]. Sexual and urinary transmission of viruses is since then of high scientific interest.

The impact of the COVID-19 pandemic on urology care has been intensively studied [10, 11]. However, it remains unclear, whether the virus is passed through the urinary tract and therefore urine might be a source of infection. SARS-CoV-2 has been detected early in urinary samples from either deceased or alive patients [2, 12]. However, SARS-CoV-2 shedding is thought to be neither prognostic nor predictive [12]. We were the first to unveil voiding frequency as a symptom in COVID-19 patients and have hypothesized a potential role of the urinary tract in COVID-19. However, it remains unclear, whether the urinary tract is involved as a result of a general inflammatory response or directly through the appearance of SARS-CoV-2 in urinary fluids which consequently might be a possible route of transmission of COVID-19 [13].

## Methods

**Fig. 1:**
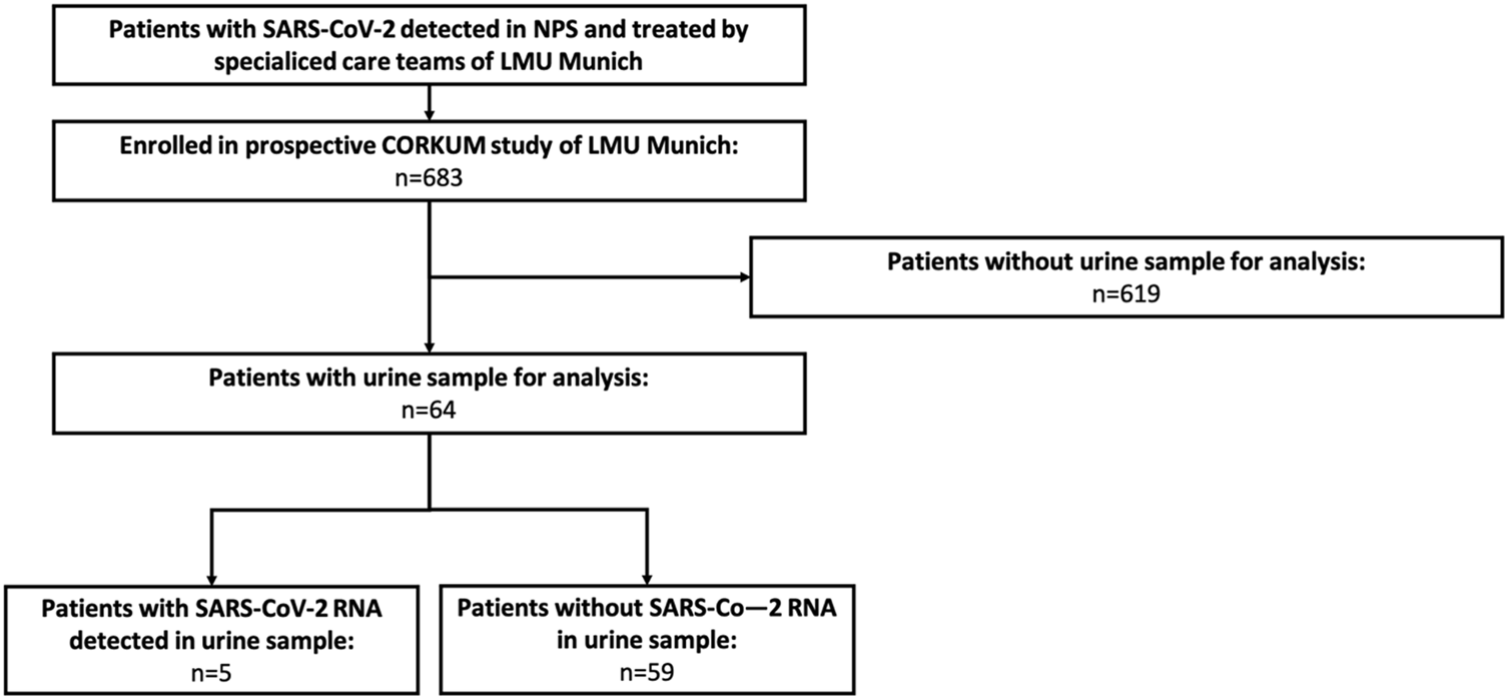
Study enrollment

With the onset of the COVID-19 pandemic, an interdisciplinary team at our institution set up a research program to investigate the novel SARS-CoV-2 and started to prospectively collect data and tissue and fluid specimen of patients with COVID-19. Prior initiation of this study the local ethics authorities had approved the project design (Reference number: 20–245). Within the first 12 months of the program, 683 patients were prospectively enrolled in this COVID-19 research program making it one of the largest national COVID-19 research programs (Fig. 1). Patients were enrolled between March 2020 and April 2021.

Clinical assessment of COVID-19 courses was performed by the WHO score as no clinical or virological evidence (0), no limitations of activity (1), limitations of activity (2), hospitalized without the need of oxygen (3), oxygen by mask or nasal tube (4), non-invasive ventilation (5), invasive ventilation (6), organ support (ECMO) (7), death (8) [14].

Immunohistochemical staining for ACE2 was performed on human tissue from surgical specimens after approval by the Ethical Review Board. Formalin-fixed, paraffin-embedded (FFPE) tissue blocks were selected for immunohistochemical analysis based on normal histology. After antigen retrieval, slides were incubated with ACE2-antibody (1:100; Abcam, Cambridge, UK). Detection was performed using the ImmPress anti-rabbit IgG polymer kit (Vector Laboratories, Burlingame, USA) according to the manufacturer's protocol. Pictures were taken at a 20 × magnification.

All tissue samples were manually scored by two independent observers (SL and MR). Evaluation was performed using a 4-level scale based on the standardized *Human Protein Atlas* (HPA) workflow [15]: 0 = Not detected (negative or weak staining in < 25% of cells); 1 = Low (weak staining in ≥ 25% of cells or moderate staining in < 25% of cells); 2 = Medium (moderate staining in ≥ 25% of cells or strong staining in < 25% of cells); or 3 = High (strong staining in ≥ 25% of cells).

Autopsies of patients who had died from Covid-19 were performed. All patients had been diagnosed with Covid-19 disease ante mortem and PCR testing for SARS-CoV-2 from postmortem nasopharyngeal swabs were in all patients RNA positive. At autopsy, an intravesical swab was collected and sent for PCR testing for SARS-CoV-2.

Urine samples were collected and stored at -20 °C until analysis. For nucleic acid extraction, the QIAsymphony DSP Virus/Pathogen Midi Kit was used for a sample volume of 400 µl on the QIAsymphony SP (Qiagen, Hilden, Germany). Amplification and quantification were performed according to the CDC protocol for the N1 target as described previously (Laboratory 1, CDC Protocol) [16, 17]. To detect inhibition of the PCR reaction, an internal positive control (IPC) was added to each sample before extraction. If this IPC was not amplifiable, the sample was diluted 1:10 with 0.9% NaCl and remeasured.

To investigate the storage stability of SARS-CoV-2 RNA in urine with respect to the conditions prevailing in this study, PCR negative donor urine was spiked with a highly positive sample (Ct value 17.2) of endotracheal aspiration. This artificial urine sample was diluted 1 × 10^–3^, 1 × 10^–4^ and 1 × 10^–5^ with 0.9% NaCl and stored at least in duplicates under the following conditions: no storage ("fresh"), 4 °C overnight, − 40 °C with 1–2 freez/thaw cycles, respectively. The Roche cobas® SARS-CoV-2 assay (Target 1) on the cobas® 6800 System (Roche Diagnostics, Basel, Switzerland) was used for analysis (Suppl. Fig. 1).

For statistical analysis, Mann–Whitney-U-Test was used. All analyses were performed by Graphpad Prism 9 (Graphpad Software, San Diego, USA). A p-value smaller than 0.05 was considered as statistically significant.

## Results

**Fig. 2:**
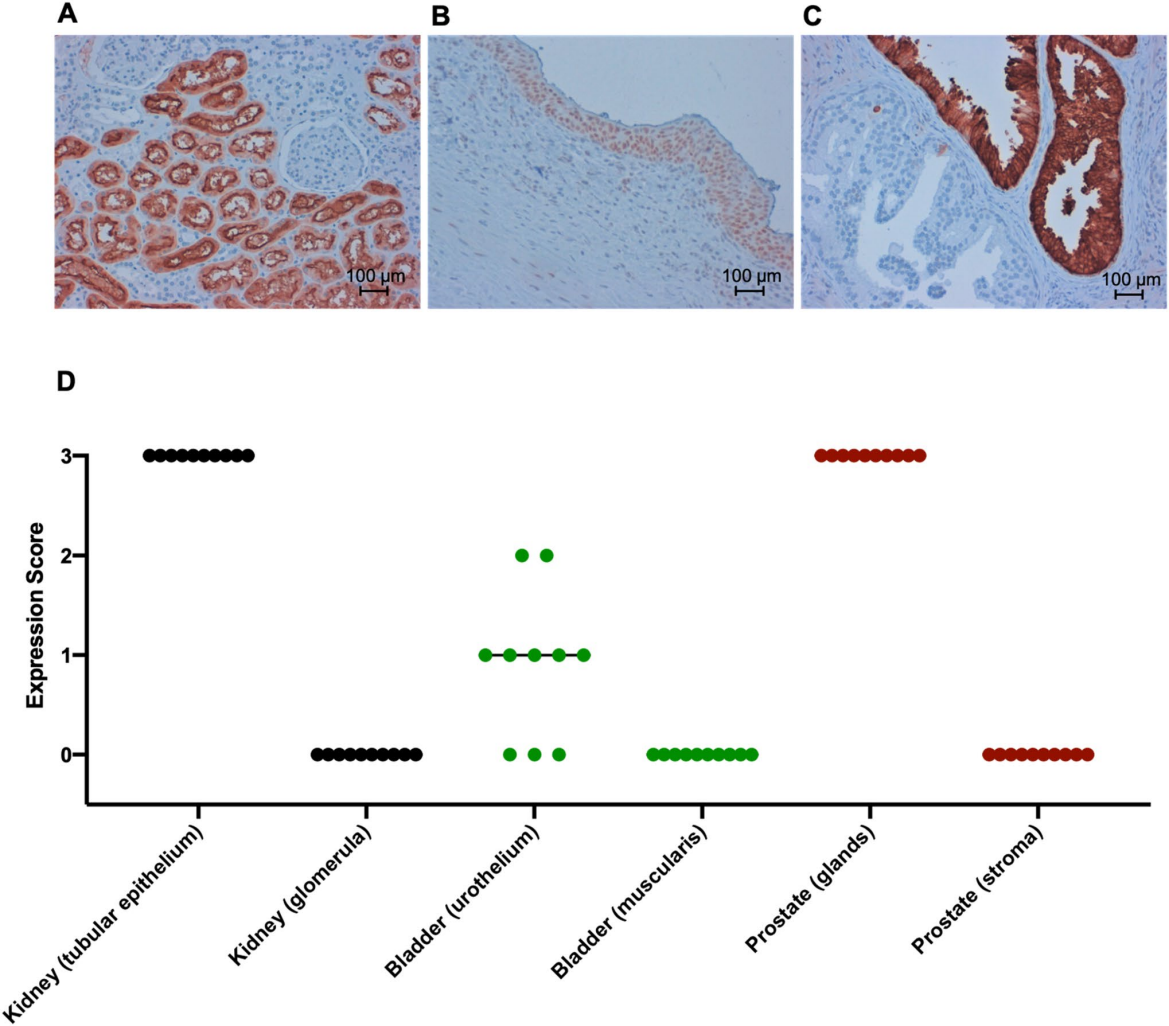
ACE-2 expression across urinary tract tissue.** A** ACE-2 was stained in kidney tissue (n = 10) via immunohistochemistry and revealed high expression in almost all epithelial cells of the proximal tubule, but not in renal corpuscles. **B** Samples from human bladders (n = 10) were stained for ACE-2 and revealed no to moderate expression in urothelial cells whereas there is no expression in the muscularis. **C** Staining for ACE-2 in prostate tissue revealed a high expression in glandular epithelium and no expression in fibromuscular stroma of the prostate. **D** ACE-2 expression across various genitourinary tissues was quantified by expression scoring for no expression (0), low expression (1), moderate expression (2) and high expression (3) in the depicted tissues

In normal kidney tissue (*n* = 10), we detected high ACE2 expression in the cytoplasma and brush border of almost all proximal tubular cells, which we quantified with an expression score of 3 as described [15]. No expression (score 0) was detected in renal corpuscles. In urinary bladder tissue (*n* = 10), no ACE2 expression was present in urothelial cells in three samples (score 0). In five samples, urothelial cells showed low ACE2 expression (score 1) and in two samples, moderate expression (score 2) was detected. In all cases, no expression of ACE2 was observed in the muscularis (score 0). In prostate tissue (*n* = 10), high expression of ACE2 (score 3) was found in the glandular epithelium of the prostate. The fibromuscular stroma of the prostate was negative (score 0; see Fig. 2).

In a second step, we analyzed prospectively collected urinary samples from patients treated at our specialized COVID-19 wards. In total, 199 urine samples from 64 patients that had been tested positive for SARS-CoV-2 in nasopharyngeal swabs were analyzed. In the initial testing, the lack of detection of IPC amplification showed complete inhibition of the PCR reaction in 24 of the urine samples. After 1:10 dilution of these samples prior to extraction and retesting, inhibition was no longer detected (for further patient characteristics see Supplementary Table 1).

**Fig. 3:**
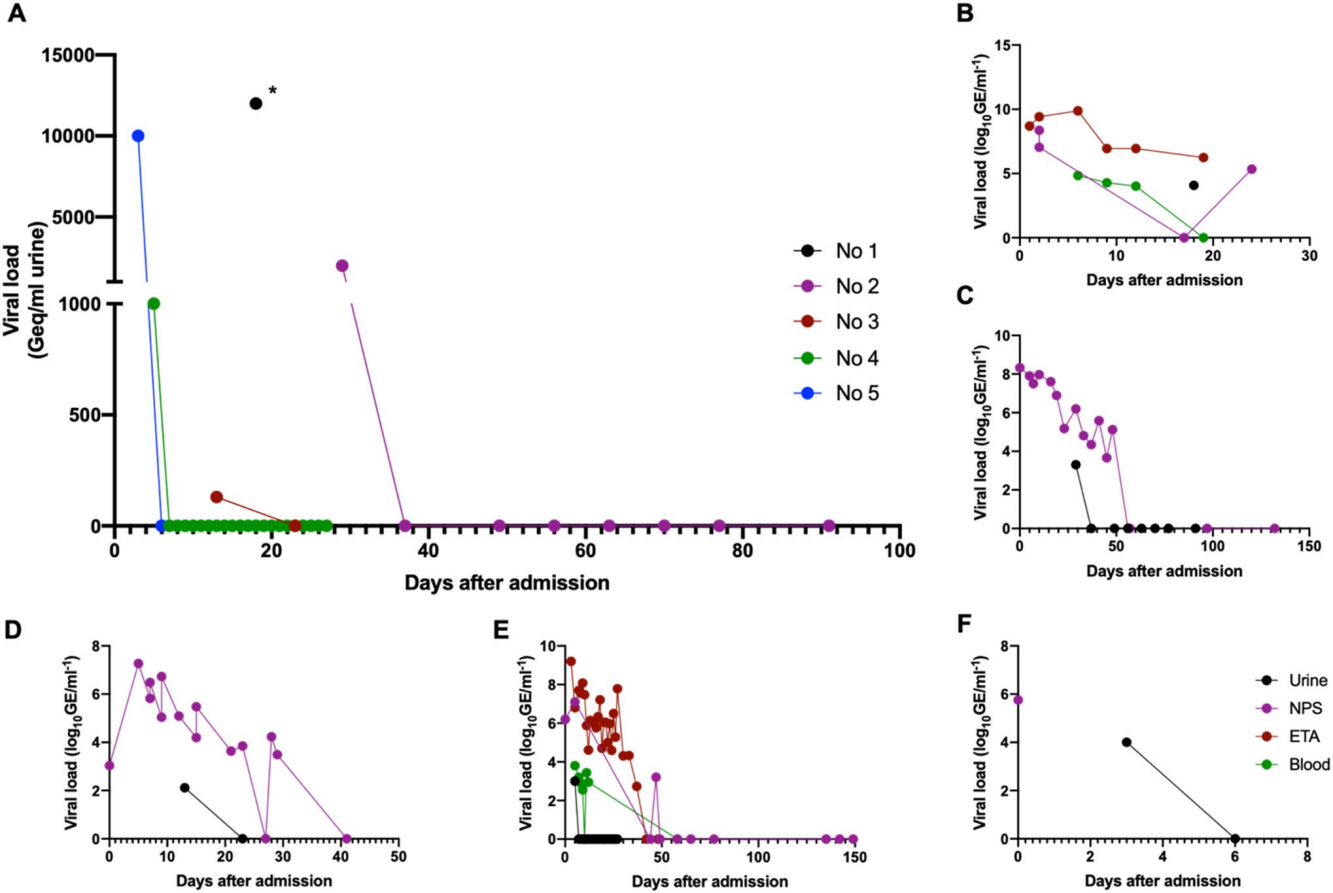
Dynamics of urinary and respiratory shedding of Severe Acute Respiratory Syndrome Virus 2 (SARS-CoV-2) RNA in COVID-19 patients.** A** Course of viral RNA load in urine samples of five patients initially tested positive for SARS-CoV-2 RNA in nasopharyngeal swaps. Viral RNA load in urine samples (urine), nasopharyngeal swab (NPS), endotracheal aspirates (ETA) or blood was analyzed for patient No 1 **B**, No 2 **C**, No 3 **D**, No 4 **E** and No 5 **(F)**. Log_10_ transformation was performed to improve understandability of the graphs. For SARS-CoV-2 negative samples we chose 1 Geq/ml to depict data more clearly. *Patient deceased on day 25 after admission, NPS: nasopharyngeal swab, ETA: endotracheal aspirates

SARS-CoV-2 RNA was detected in the urine of five patients. We observed viral RNA loads of 130 Geq/ml, 1000 Geq/ml, 2,000 Geq/ml, 10,000 Geq/ml and 12,000 Geq/ml in urines of those five patients, respectively. SARS-CoV-2 RNA was always detected between day 3 and 29. Interestingly, it was always the first sample taken from the respective patients that was positive for RNA. Urines (*n* = 194) of patients tested negative were collected between day 1 and day 63 after admission. Analysis of viral RNA load in other specimen as nasopharyngeal swabs, endotracheal aspirates or blood revealed higher viral RNA loads than in urine samples prior or after this respective measurement (Fig. 3). Additionally, intravesical swabs in patients deceased with COVID-19 revealed positive RNA results in 3 out of 4 patients.

**Fig. 4:**
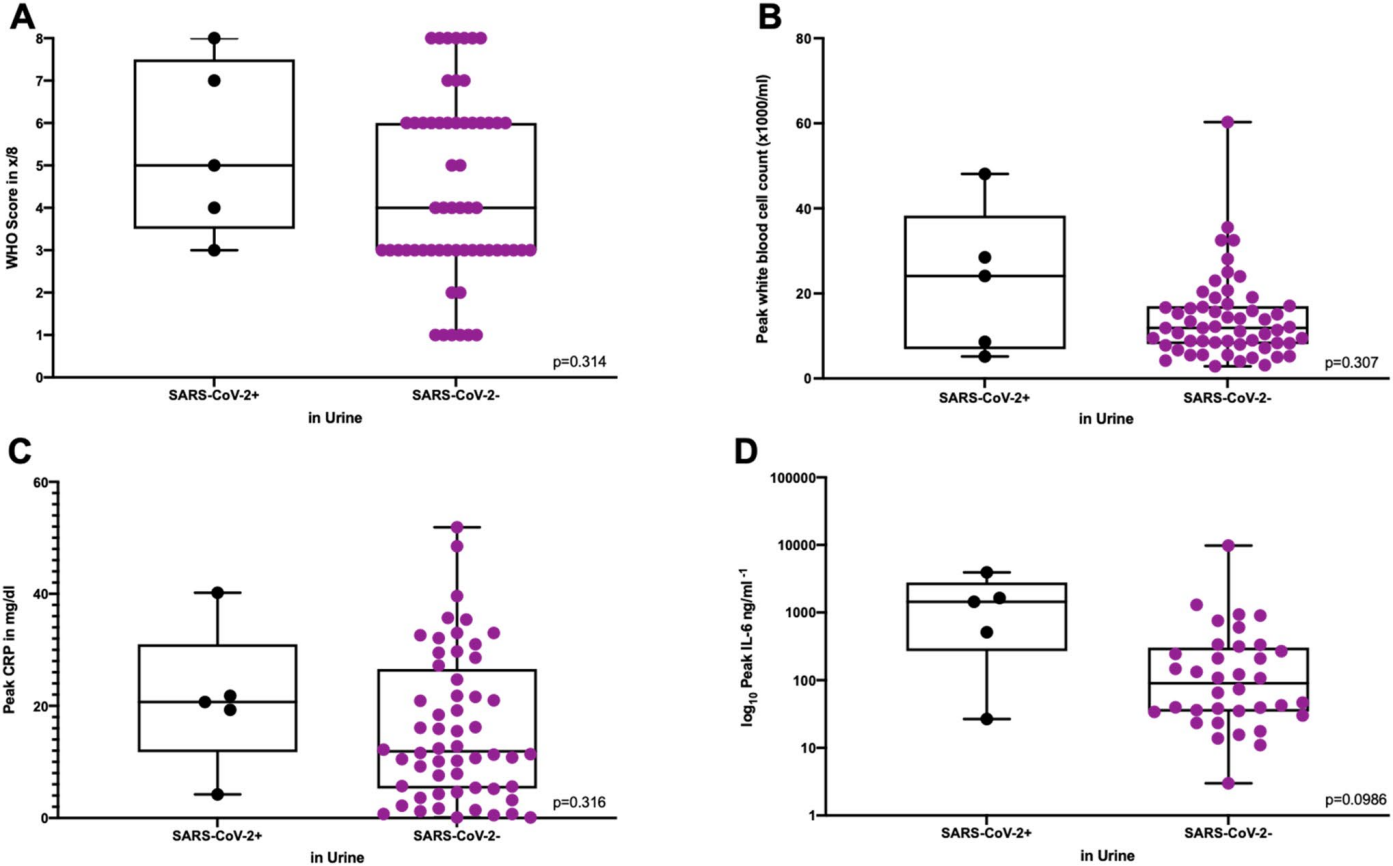
Clinical outcomes of urinary SARS-CoV-2 RNA positive and SARS-CoV-2 RNA negative patients.** A **COVID-19 severity was calculated with the WHO score as described for patients with SARS-CoV-2 RNA detected in urine samples (SARS-CoV-2 +) and without detection of SARS-CoV-2 RNA (SARS-CoV-2-). **B** The highest white blood cell count during treatment at one of the COVID-19 wards was defined as the peak white blood cell count for the respective groups. Peak CRP **C** and peak IL-6 **D** were defined accordingly

Patients with and without SARS-CoV-2 in urine were analyzed for differences in clinical outcomes. The worse clinical outcome in patients with positively tested urine was death (*n* = 1), ECMO (*n* = 1), non-invasive ventilation (*n* = 1), oxygen by mask or nasal tube (*n* = 1) and hospitalized without need for oxygen (*n* = 1). The highest WHO score during treatment was not significantly different between patients with SARS-CoV-2 RNA detected in the urine (median 5, range 3–8) and patients without (median 4, range 1–8, *p* = 0.314). Inflammatory markers as peak white blood cell count (median 24.1 × 1000/ml, range 5.19–48.1 versus median 11.9 × 1000/ml, range 2.9–60.3, *p* = 0.307), peak CRP (median 20.7 mg/dl, 4.2–40.2 versus median 11.9 mg/dl, range 0.1–51.9, *p* = 0.316) and peak IL-6 levels (median: 1442 ng/ml, range 26.7–3918 versus median 140 ng/ml, range 3.0–11,041, *p* = 0.099) were not significantly different between both groups (Fig. 4).

Stability of SARS-CoV-2 RNA in urinary samples was tested to exclude errors generated by storage conditions. After incubation and cycles of freeze/ thaw of an artificially spiked urine sample as described above no differences in *C*_*t*_-values were observed between the samples (*p* = 0.889, Supplementary Fig. 1 and Supplementary Table 1).

## Discussion

This is one of the largest and most comprehensive studies to analyze SARS-CoV-2 RNA in the urine specimen and to investigate possible transmission by urinary tissue.

### ACE2 expression in tissues of the urinary tract

ACE2 expression has previously been observed in the kidney mainly in the proximal tubular cells [18, 19]. In a study with a publicly available single-cell RNA-sequencing data set, coexpression of ACE2 and TMPRSS2 was seen in prostate cells [20]. Although known for years [21], ACE-2 and its expression especially in the kidney has gained traction during the rise of the COVID-19 pandemic and through increased knowledge of the replication mechanism of SARS-CoV-2 [22]. We observed high expression levels in the tubular epithelium of the kidney and in prostate glands, as well as intermediate expression in urothelial cells of the bladder. Our data confirms previous knowledge of ACE-2 expression and reveals its expression throughout the urinary tract.

### Stability of SARS-CoV-2 in urine and impact of urine on SARS-CoV-2 PCR results

As we artificially created SARS-CoV-2 infected urine, we comprehensively analyzed stability and interference of urine on results in SARS-CoV-2 RNA detection. SARS-CoV-2 RNA detection rates are stable under storage conditions and, therefore, preanalytical interference can be excluded. However, we observed inhibition of PCR reactions in some urine samples of patients with urinary tract infection.

### Detection of SARS-CoV-2 RNA in urine of COVID-19 patients

Previous groups have detected SARS-CoV-2 RNA in urine samples. Thereby, SARS-CoV-2 RNA was detected in 4.5% of 533 patients across 39 studies. However, the analyzed cohorts were highly heterogenous and differed in sampling technique and origin. Some samples were derived from already dead patients [23]. As we hypothesize that there is a high risk of bias and analytic errors, we performed standardized analysis through our virology department. We only included alive patients and observed SARS-CoV-2 RNA in 2.5% of all samples and in 7.8% of all patients.

### Correlation of urinary shedding and COVID-19 severity

We have not detected any correlation between patients being tested positive in urine for SARS-CoV-2 and clinical outcomes in our cohort. Previously, it has been demonstrated that respiratory SARS-CoV-2 shedding and high IL-6 and CRP levels revealing a protracted inflammatory response was associated with severe disease [24]. Our analysis of IL-6 and CRP peak levels revealed no difference between patients with and without detection of SARS-CoV-2 RNA in the urine. Other studies observed urinary shedding mainly in moderate and severe COVID-19 cases [23] comparable to our study. However, we did not observe a significant difference in disease courses in our cohort.

Interestingly, patients who had died because of a SARS-CoV-2 infection had high rates of viral RNA in the bladder compared to surviving patients. We hypothesize that this effect is due to anti-mortem and post-mortem autolysis or necrosis of the tissue and degradation of epithelial barriers. Therefore, we dispute previous findings of positive PCR results from urine samples in this population.

### SARS-CoV-2 transmission via urine and urinary tract

With only low concentrations of SARS-CoV-2 RNA in urine, the transmission of SARS-CoV-2 via urinary tissues seems negligible, as airway transmission has revealed dependency on viral RNA load [25]. Therefore, it appears to be highly unlikely, that urine transmission is a relevant risk for transmission in patients tested negative in NPS or ETA. SARS-CoV-2 NPS testing, therefore, gives sufficient information to protect urologists from infections during cystoscopy or other endo-urologic procedures under the adequate hygienic conditions.

### Limitations

Our study is limited by the study design and sample size. Patients were prospectively enrolled in our follow-up program, but analysis of urines was performed retrospectively. Therefore, we performed stability testing. A systematic bias due to sample stability can be excluded based on our results. In addition, SARS-CoV-2 viral RNA load was quantified and not the infectious virus. For other viruses as for instance the Ebola virus a prolonged viral RNA excretion has been demonstrated without virus shedding measured by virus isolation [26]. As only five urinary samples were positive, subgroup analysis and clinical outcome comparison are limited. We also have included mainly patients with moderate to severe symptoms of COVID-19, as they have been treated at our specialized wards. However, this is one of the largest and most comprehensive analysis of urinary shedding and implications for transmission.

## Conclusion

ACE-2 is expressed through the urinary tract and provides thereby a potential route of transmission via the genitourinary tract. SARS-CoV-2 RNA can be detected in the urine of a minority of patients with COVID-19, but higher levels are seen in other specimen such as nasopharyngeal swabs. As detection of SARS-CoV-2 RNA seems not to be prognostic and viral shedding is observed with lower concentrations than other potential routes of transmission as airways, a relevant risk of urinary transmission compared to transmission via aerosol and direct contact routes is highly unlikely.

## Supplementary Information

Below is the link to the electronic supplementary material.

Supplementary file1 (DOCX 44 KB)

## Data Availability

Data is available from the corresponding author upon request.

## Code availability

No statistical code or only trivial code has been used.
