# Supplementary material for: Dynamics of urinary and respiratory shedding of Severe acute respiratory syndrome virus 2 (SARS-CoV-2) RNA excludes urine as a relevant source of viral transmission
Source: Infection. 2021 Oct 30;50(3):635–42. doi: 10.1007/s15010-021-01724-4 (PMC8556791; doi:10.1007/s15010-021-01724-4)
Supplement: Supplementary file 1 — Supplementary file1 (DOCX 44 KB) [file 15010_2021_1724_MOESM1_ESM.docx]

**Supplementary material**

**Supplementary table 1: Patient characteristics**

|  | **SARS-CoV-2 in urine**  (n=5) | | **SARS-CoV-2 negativ**  (n=59) | | **p-value** |
| --- | --- | --- | --- | --- | --- |
| **Age**  Median  Range | 65  22-76 | | 60  20-82 | | 0.831 |
|  |  |  |  |  |  |
|  | **n** | **%** | **n** | **%** |  |
| **Gender**  Male  Female | 2  3 | 40.0  60.0 | 44  15 | 74.6  25.4 | 0.099 |
| **Survival status**  Alive  Deceased in hospital | 4  1 | 80.0  20.0 | 52  7 | 88.1  11.9 | 0.597 |

**Supplementary figure 1: Stability of SARS-CoV-2 RNA in urinary samples**

PCR negative donor urine (control) was spiked with endotracheal secret with a known Cp value of 17.2 and diluted 1x10^-3^, 1x10^-4^ and 1x10^-5^ with 0.9% NaCl. RT-PCR was performed either directly (fresh), after overnight incubation at 4°C or after one or two cycles of freezing at -40°C and thawing. At least duplicates were performed, and medians and ranges are depicted.

Abbr.: Cp: crossing point
